# Eukaryotes Are a Holophyletic Group of Polyphyletic Origin

**DOI:** 10.3389/fmicb.2020.01380

**Published:** 2020-07-02

**Authors:** Josip Skejo, Damjan Franjević

**Affiliations:** ^1^Institute of Molecular Evolution, Heinrich–Heine University Düsseldorf, Düsseldorf, Germany; ^2^Evolution Lab, Division of Zoology, Department of Biology, Faculty of Science, University of Zagreb, Zagreb, Croatia

**Keywords:** Eukaryomorpha, archaea, alphaproteobacteria, eukaryogenesis, lichens, hybridization, symbiogenesis, paraphyly

## Introduction

All living beings can be assigned to one of the three domains of life (Woese et al., 1990; Williams et al., 2013), all of which are monophyletic (Doolittle, 2014). Two prokaryotic domains, Archaea and Bacteria, are characterized by the lack of intercellular compartments (Martin, 1999; McInerney et al., 2014), whereas eukaryotes, characterized by the complexity of cellular structures and life cycle, originated via symbiogenesis of an archaeal host and a bacterial endosymbiont i.e. proto-mitochondrion (Mereschkowsky, 1905; Zimorski et al., 2014; Muñoz-Gómez et al., 2017; Roger et al., 2017). With millions of described species (Costello et al., 2013; Adl et al., 2019), eukaryotes are morphologically the most diverse of the three groups bearing symbiogenesis as the hallmark of their evolutionary origin (Wallin, 1927; Margulis, 1991). Symbiogenesis has always been a common phenomenon in the eukaryotic evolution (McFadden, 2001; Nowack and Melkonian, 2010; Bonfante and Desirò, 2017). Nevertheless, there are still many unanswered questions regarding the prokaryotes that participated in eukaryogenesis. The true evolutionary position of eukaryotes is hence the subject of continuing debates and it has still not been widely agreed if eukaryotes represent a separate domain (Williams et al., 2013; Doolittle, 2020). Alphaproteobacteria is known to be the ancestor of mitochondria (Roger et al., 2017). However, our understanding of the archaeal lineage that gave rise to the eukaryotic nuclear genome is still insufficient. Asgard archaea, which were recently identified based on metagenome-assembled sequences (Spang et al., 2015; Seitz et al., 2016; Zaremba-Niedzwiedzka et al., 2017; MacLeod et al., 2019), possess eukaryotic signature proteins (ESPs) involved in cytoskeleton regulation (Akil and Robinson, 2018; Akil et al., 2019), and are being cultivated now (Imachi et al., 2020). The first photographed member of Asgard is known under the name “Candidatus *Prometheoarchaeum syntrophicum*,” and it does not exhibit eukaryotic features (such as the presence of mitochondrion, nucleus, endoplasmic reticulum, or sexual reproduction), but rather exhibits typical prokaryotic features, such as small size, spherical (coccoid) body, and lack of organelles (Imachi et al., 2020). Recently, Fournier and Poole (2018) presented a taxonomic view in which Asgard represented the main eukaryotic ancestor (parent) and were, along with eukaryotes, united into a “monophyletic” group named Eukaryomorpha. The aim of this opinion manuscript is to debate this newly introduced term. We briefly review the meaning of the terms “monophyletic” and “polyphyletic,” and we draw attention to the bacterial contribution to eukaryogenesis.

## Paraphyletic Means Monophyletic

**Figure 1:**
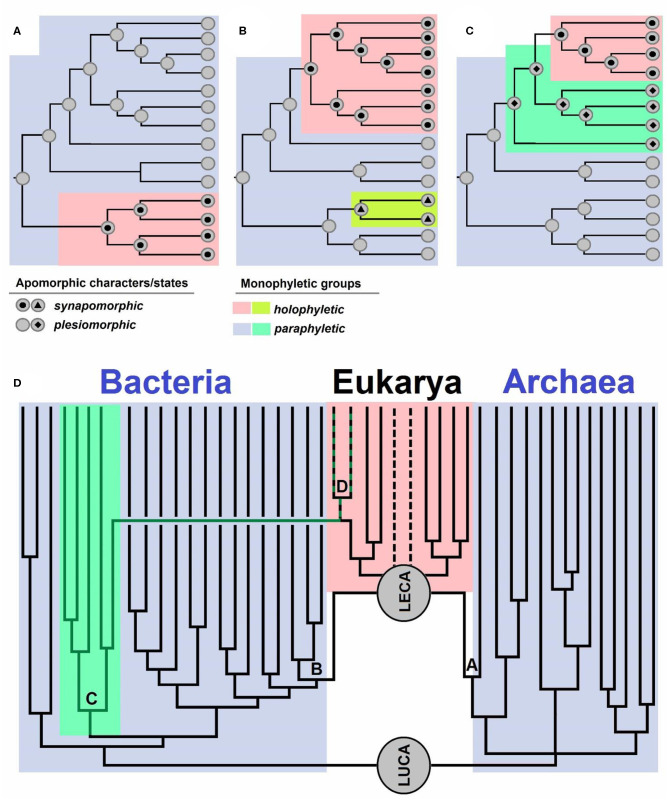
**(A–C)** Schematic overview of apomorphic characters and states. Synapomorphy is shared by all the members of a group descending from a single ancestor. Plesiomorphy is ancestral and as such not present in all the members of a group. Monophyletic groups are characterized by apomorphies: synapomorphies in holophyletic or plesiomorphies in paraphyletic groups. Topology of the cladograms shown in **(A–C)** is the same, but the distributions of characters and their states are different. Case **(A)** shows a paraphyletic group from which a holophyletic descendant is excluded. Case **(B)** shows a paraphyletic group with two holophyletic descendants excluded. Case **(C)** shows two paraphyletic groups and a holophyletic group. **(D)** Schematic representation of the evolution of life from its last common ancestor (LUCA), which gave rise to Bacteria and Archaea [the diversity is simplified, and descendants of archeal trichotomy represent Euryarchaeota, TACK+Asgard (Asgard is sister to LECA)]. LECA is the last eukaryotic common ancestor, which originated via a polyphyletic event: symbiogenesis of an archaeon **(A)** which gave rise to nuclei, and Bacteria **(B)**, specifically Alphaproteobacteria, which gave rise to mitochondria. Cyanobacteria **(C)** are a group of bacteria from which the primary plastid **(D)** originated. The dotted lines represent groups with uncertain positions within Eukaryotes.

Evolutionary biologists use the term “monophyly” in various ways (see e.g., Envall, 2008), just as Hennig (1950, 1966), the creator of the term, originally did, which has hitherto ensued a lot of confusion (Envall, 2008). In this opinion, we use the term “monophyletic” only for groups with a single definable ancestor, meaning that paraphyletic groups are also considered as monophyletic. Each taxonomic group can be characterized by either having a shared (single) ancestor—“monophyletic group” *or* having numerous ancestors—“polyphyletic group” (Hennig, 1950, 1966). Polyphyletic groups are not taxonomically desirable, and traditionally, characters shared by members of such a group represent *homoplasies* (analogies), i.e., traits that evolved independently in similar environments on account of similar selective advantages (Wake et al., 2011). A historical error occurred when Hennig (1950, 1966) defined two groups, monophyletic and paraphyletic, based on the inclusion of all descendants of a given ancestor. If all the descendants of a given ancestor belonged to one group, it was regarded as a *monophyletic* group, and if this was not the case, it was regarded as a *paraphyletic* group (Hennig, 1950, 1966). Missing from such definition was the distinction between a group with a single ancestor and a group that includes all the descendants of an ancestor, which were both defined as monophyletic by Hennig (1950, 1966). Ashlock (1971, 1972, 1974, 1979) noticed the erratum and introduced the term “holophyletic group,” referring to a monophyletic group that includes all the descendants of an ancestor. Therefore, a “paraphyletic group” *is* a monophyletic one that does not include all the descendants of an ancestor (Figures 1A–C).

Well-known examples of holophyletic groups are mammals (descendants of Therapsida), snakes (descendants of earless and legless lizards), birds (descendants of Dinosauria), modern amphibians, tetrapods (land vertebrates, descendants of fish), jawed vertebrates, bilaterians (bilaterally symmetric animals), animals, and eukaryotes (Pough et al., 1999; Nielsen, 2012; Doolittle, 2014). Examples of paraphyletic groups are reptiles or amniotes (whose descendants are mammals and birds), amphibians (a group including Lissamphibia and extinct amphibians whose descendants are reptiles), sarcopterygians (whose descendants are tetrapods), fish (Pisces) (as they include all vertebrates excluding those inhabiting land), jawless fish (lampreys, hagfish, and extinct groups related to them, whose descendants are also jawed fish), bryophytes in wider sense (as land plants are their descendants), streptophytes (stonewort and relatives, if plants are excluded), archaeplastids (as secondary plastids of SAR and euglenoids are not considered to be archaeplastid members anymore), cyanobacteria (because plastids are regarded as organelles, not cyanobacteria anymore), prokaryotes (because eukaryotes are excluded), Archaea (because the nucleus is not regarded to be an archeon anymore), and Bacteria (because mitochondria are not regarded as Alphaproteobacteria anymore; Pough et al., 1999; Nielsen, 2012; Doolittle, 2014).

If we ignore the presence of mitochondria and existence of lateral gene transfer from bacteria to the eukaryotic host, the origin of the eukaryotic nucleus could be compared to the origin of mammals and birds within amniotes, as described in Fournier and Poole (2018). However, the origin of eukaryotes is not comparable to the origin of these groups, and the bacterial contribution to eukaryogenesis should not be neglected. Eukaryotes are of polyphyletic origin, as their ancestor, LECA, sits on both branches of life—the archaeal (Asgard) and the bacterial branch (Alphaproteobacteria).

## Polyphyletic, Reticulated Events in Evolution

Well-established examples of natural polyphyletic events include lateral gene transfer (LGT) in prokaryotes (Nelson-Sathi et al., 2015), symbiogenesis in prokaryotes and eukaryotes (biofilms, endosymbiosis, ectosymbiosis, etc.; e.g., Vogels et al., 1980; López et al., 2010; Naumann et al., 2010), and sexual reproduction in eukaryotes (Speijer et al., 2015). Genes can also be of polyphyletic origin; those genes are known as chimeric genes (e.g., Méheust et al., 2018). Polyphyletic origin is an evolutionary event in which two lineages (individuals, populations, or species) merge into a single, “chimeric” lineage. A lineage of polyphyletic origin should not be united with any of its ancestors in an attempt to form a higher monophyletic group, as it will not result in such. Even though eukaryotes are a monophyletic and holophyletic group by definition, they are of polyphyletic origin because of the very nature of their ancestor's, LECA's origin. Today, the polyphyletic origin of eukaryotes is a well-supported scientific theory. Eukaryotic (syn)apomorphies are the traits of eukaryotic complexity: nuclei, mitochondria, Golgi apparatus, endoplasmic reticulum, and sexual reproduction (Koonin, 2010; Koumandou et al., 2013; Garg and Martin, 2016; Doolittle, 2020).

Eukaryogenesis is not a unique example of polyphyletic origin of a monophyletic group. Other such events are widely dispersed in the tree of life. Known examples are hybrid species, which originated via hybridization of two species, usually (but not always) from the same genus (Seehausen, 2004; Grant and Grant, 2008; Meier et al., 2017). *Homo sapiens* is an example of such species. It is a hybrid between *H. heidelbergensis, H. neanderthalensis*, and Denisovians (Sankararaman et al., 2016). The Jutland bow-winged grasshopper (*Chorthippus jutlandica*) is a unique species which originated from the hybridization of *C. brunneus* and *C. biguttulus* in Denmark (Gottsberger, 2007). Domestic wheat is a hybrid between species belonging to the genera *Triticum* and *Aegilops* (Ozkan et al., 2001). There are even examples of one of the ancestral species being extinct, but its mitochondrial genome still being present, which is called a ghost lineage (Recuero et al., 2014). There is no example of a natural monophyletic group that could be composed of any of the aforementioned species and one of its parents, as is the case with Eukaryotes, Asgard, and Eukaryomorpha.

Lichens not only gave rise to the concept of symbiosis (de Bary, 1879), but they are also the classical example of organisms that originated by symbiogenesis (Lutzoni and Miadlikowska, 2009). Lichen species are composed of mycobionts (Ascomycota and/or Basidiomycota) and photobionts (Chlorophyta or Cyanobacteria; Lutzoni and Miadlikowska, 2009; Spribille et al., 2016; Tuovinen et al., 2019). Symbiosis is species-specific (Lindsay, 1856), co-dependent, and the symbionts usually cannot survive outside the lichen. Lichens are an example of a polyphyletic group with multiple polyphyletic origins. Relatives of lichen-forming green algae (symbiont lineages) should not be designated as “Lichenomorpha,” even though they represent one of the constituent evolutionary lineages that gave rise to lichens. Cyanobacteria should not be designated as “Plastidomorpha,” despite the fact that this group contains the ancestors of plastids. The case of Archaeplastida (primary photosynthetic eukaryotes) is an interesting one and should be addressed in a separate essay. The supergroup originated via plastidogenesis, an anastomosis between cyanobacteria and eukaryotes; and has since contributed to many anastomoses (secondary endosymbioses) in the eukaryotic tree (McFadden, 2001). The origin of the plastid may be comparable to the origin of mitochondria, however probably only to a certain extent, because of the complexity of the archaeplastidian eukaryotic parent.

## Bacterial Contribution to Eukaryogenesis Should not be Neglected

Bacteria (mainly Alphaproteobacteria, but others as well) are as important as Archaea in eukaryogenesis. Mitochondria are of alphaproteobacterial origin, nuclei of chimeric (archaeal and bacterial), and plastids of cyanobacterial origin. The strongest signals in eukaryotic genomes are, indeed, proteobacterial, archaeal, and cyanobacterial (Pisani et al., 2007; Ku et al., 2015). Because of the combination of archaeal and bacterial features exhibited by eukaryotes, they should not be assigned to a higher taxon along with any of their ancestors.

Eukaryotes exhibit a unique mixture of prokaryotic features, most of which can be traced back to either Archaea or Bacteria. Unlike prokaryotes, eukaryotes do not exchange genes via LGT, but by sexual reproduction (Ku et al., 2015). An archaeon is known to have been the host of the eukaryote-forming endosymbiosis, contributing genetic machinery and ribosomal DNA (Esser et al., 2004; Thiergart et al., 2012; Gould et al., 2016). There is an interesting hypothesis stating that eukaryotic membranes originated from bacterial vesicle secretion (Gould et al., 2016). The genes encoded in the nucleus are as bacterial as they are archaeal. A larger part of the eukaryotic genome has bacterial homologs (Esser et al., 2004; Brueckner and Martin, 2020) that most likely originated from the EGT (endosymbiotic gene transfer) with the proto-mitochondrion ancestor (Brueckner and Martin, 2020), whereas archaeal genes are less numerous in eukaryotic genome, but also important (Pisani et al., 2007; Brueckner and Martin, 2020). The origin of mitochondrion was a prerequisite for the existence of sexual reproduction and meiosis. These processes required large amounts of energy (ATP), and no known prokaryotic cell is able to produce such amount of ATP (Garg and Martin, 2016). Some authors still dispute the uniqueness of eukaryogenesis and the importance of mitochondria in the definition of eukaryotes (e.g., Booth and Doolittle, 2015; Lynch and Marinov, 2016).

We think that the bacterial contribution to eukaryogenesis should not be neglected in view of the facts that: (1) mitochondria, whose presence is a eukaryotic synapomorphy, represents the true descendant of Alphaproteobacteria, (2) most of the eukaryotic nuclear DNA originated via gene transfer from bacteria, and (3) all eukaryotic membranes may be of bacterial origin.

## Concluding Thoughts

Because of the polyphyletic origin of the eukaryotic monophylum, eukaryogenesis within prokaryotes is not comparable with mammal origin within paraphyletic reptiles. Both synapomorphies and plesiomorphies represent apomorphies and are indeed suitable for defining monophyletic (holophyletic and paraphyletic) groups. Alphaproteobacteria (Bacteria) and Asgard (Archaea) are the ancestors of LECA (the Last Eukaryotic Common Ancestor). The presence of ESPs in Asgard does not dispute the polyphyletic origin of eukaryotes; it only further corroborates it. “Candidatus *Prometheoarchaeum syntrophicum*” is the closest relative to eukaryotes and the only Asgard with available microscopy data. This newly discovered species has a prokaryotic cell organization and does not exhibit features of eukaryotic complexity (nucleus, mitochondrion, meiotic cycle), and thus, it does not belong to Eukaryomorpha.

Along with Cyanobacteria, non-photosynthetic eukaryotes are the ancestors of the primary photosynthetic eukaryotes (archaeplastidians). Non-photosynthetic eukaryotes are not the ancestors of plastids, hence LECA is not the only ancestor of the extant eukaryotic diversity. Eukaryotes are monophyletic by definition, as they have a single ancestor, LECA. They are also holophyletic as all LECA's descendants belong to the same group. They are polyphyletic as well since they exhibit numerous symbioses and anastomoses in the tree of life.

Symbiogenesis will always be one of the major forces driving eukaryotic evolution. A group of polyphyletic origin, such as eukaryotes, should not be assigned to a higher taxon that contains its single parent, as is the case with Eukaryomorpha.

## Author Contributions

JS contributed to conceptualization, investigation, data curation, writing (original draft), and visualization. DF was responsible for the validation, resources, writing (review and editing), and supervision. All authors contributed to the article and approved the submitted version.

## Conflict of Interest

The authors declare that the research was conducted in the absence of any commercial or financial relationships that could be construed as a potential conflict of interest.
